# XBP1S, a BMP2-inducible transcription factor, accelerates endochondral bone growth by activating GEP growth factor

**DOI:** 10.1111/jcmm.12261

**Published:** 2014-03-18

**Authors:** Feng-Jin Guo, Zhangyuan Xiong, Xiaofeng Han, Chuanju Liu, Yanna Liu, Rong Jiang, Peng Zhang

**Affiliations:** a Department of Cell Biology and Genetics, Core Facility of Development Biology, Chongqing Medical UniversityChongqing, China; b Departments of Orthopaedic Surgery and Cell Biology, New York University School of MedicineNew York, NY, USA; c Laboratory of Stem Cells and Tissue Engineering, Chongqing Medical UniversityChongqing, China

**Keywords:** X-box binding protein 1 spliced (XBP1S), chondrogenesis, GEP, BMP2, unfolded protein response

## Abstract

We previously reported that transcription factor XBP1S binds to RUNX2 and enhances chondrocyte hypertrophy through acting as a cofactor of RUNX2. Herein, we report that XBP1S is a key downstream molecule of BMP2 and is required for BMP2-mediated chondrocyte differentiation. XBP1S is up-regulated during chondrocyte differentiation and demonstrates the temporal and spatial expression pattern during skeletal development. XBP1S stimulates chondrocyte differentiation from mesenchymal stem cells *in vitro* and endochondral ossification *ex vivo*. In addition, XBP1S activates granulin-epithelin precursor (GEP), a growth factor known to stimulate chondrogenesis, and endogenous GEP is required, at least in part, for XBP1S-stimulated chondrocyte hypertrophy, mineralization and endochondral bone formation. Furthermore, XBP1S enhances GEP-stimulated chondrogenesis and endochondral bone formation. Collectively, these findings demonstrate that XBP1S, a BMP2-inducible transcription factor, positively regulates endochondral bone formation by activating GEP chondrogenic growth factor.

## Introduction

During foetal development of the mammalian skeletal system, the majority of bones form through a process of endochondral ossification. Chondrocytes in the primary centre of ossification begin to grow. Elaborate chondrogenesis is controlled exquisitely by cellular interactions with the growth factors, surrounding matrix proteins and other environmental factors that mediate cellular signalling pathways and transcription of specific genes in a temporal-spatial manner [1–3]. Production of and response to different growth factors are observed at all times, such as transforming growth factor-β (TGF-β) superfamily and bone morphogenic protein (BMP) subfamily. BMP2 is one of the most important cytokines and plays several important roles in a variety of cellular functions ranging from embryogenesis, cell growth, and differentiation to bone development and the repair of bone fractures [4,5]. Jang *et al*. [6] reported that BMP2 activates UPR transducers, such as PERK (PKR-like ER-resistant kinase), OASIS and ATF6 (activating transcription factor 6). BMP2 induces osteoblast differentiation through Runx2-dependent ATF6 expression, which directly regulates osteocalcin transcription. OASIS [7], a member of the CREB/ATF family, activates the transcription of Col1a1 through an unfolded protein response element (UPRE)-like sequence in the osteoblast-specific Col1a1 promoter region. The expression of OASIS in osteoblasts is induced by BMP2, the signalling of which is required for bone formation.

Human XBP1 (X-box-binding protein 1) is a signalling molecule downstream of IRE1 in the IRE1-XBP1 pathway of the UPR and participates in IRE1α-mediated UPR signal transmission. In eukaryotic cell, IRE1 is activated by ER stress and subsequently processes XBP1 mRNA to generate the spliced form of XBP1 protein (XBP1S). XBP1 exists in two forms: XBP1S and XBP1U (XBP1 unspliced form) isoforms [8–10]. Tohmonda [11] reported that inositol-requiring protein 1α (IRE1α), one of the most crucial UPR mediators, and its target transcription factor XBP1 is essential for BMP2-induced osteoblast differentiation. Osterix (Osx, a transcription factor that is indispensible for bone formation) is a target gene of XBP1. The IRE1α-XBP1 pathway is involved in osteoblast differentiation through promoting Osterix transcription by XBP1. Although there is some evidence that XBP1 plays an important role in the control of cell proliferation and the differentiation of numerous types of cells and tissues, including adipogenesis, myelomapathogenesis, skeletal muscle myotubes and dendritic cells in ER stress [12–15], little is known about the modulation and physiological significance of XBP1S in chondrocyte development and bone formation. Specifically, the molecular mechanism by which XBP1S regulates chondrogenesis also remains unknown.

Granulin-epithelin precursor (GEP), also referred to as pro-granulin, acrogranin, was first purified as a growth factor from conditioned tissue culture media. Granulin-epithelin precursor is a 593 amino acid secreted glycoprotein with an apparent molecular weight of 80 kD [16–18]. Granulin-epithelin precursor is secreted in an intact form and undergoes proteolysis, leading to the release of its constituent peptides, the granulins [19,20]. Granulin-epithelin precursor contains 7.5 repeats of a cysteine-rich motif (CX_5–6_CX_5_CCX_8_CCX_6_CCXD X_2_HCCPX_4_CX_5–6_C) in the order P-G-F-B-A-C-D-E, where A-G are full repeats and P is a half motif. Granulin-epithelin precursor is remarkably expressed in rapidly cycling epithelial cells, in chondrocytes [21–23], in the immune system cells, in neurons and in some human cancers [24–27]. Increasing evidence has implicated GEP in the regulation of differentiation, development and pathological processes. It has been isolated as a differentially expressed gene from macrophage development [28], skeletal muscle differentiation [29] and synovium (morphopathogenesis) in rheumatoid arthritis and osteoarthritis [22,30]. Granulin-epithelin precursor was also shown to be a critical mediator of wound response and tissue repair [31,32]. We previously reported that GEP regulates chondrocyte differentiation and endochondral bone formation, and cartilage repair through Erk1/2 signalling and its target gene, including JunB transcription factor [21].

In this study, we attempt to determine whether XBP1S is essential for skeletal development using both *in vitro* and *in vivo* approaches. Second, we studied its upstream and downstream molecules during chondrogenesis, as well as its molecular mechanisms by which XBP1S regulates chondrogenesis. Our results support a novel role of XBP1S, a key downstream molecule of BMP2 in the control of chondrogenesis and endochondral bone growth through activating GEP growth factor.

## Materials and methods

### Plasmids and adenoviruses

To generate pGL3-XBP1-luc reporter plasmid, the corresponding segments were amplified using PCR with the following primers: 5′-GTCACGCGACGCTGGCCAATCGCGG AGGGCCACGAC-3′ and 5′-GTCGTGGCCCTCCGCGATTGGCCAGCGTCGCGTGAC-3′ for pGL3-XBP1-luc; PCR products were inserted into the pGL3 vector.

To generate XBP1S small interfering RNA (siRNA) expression constructs, siRNA corresponding to the coding sequence of the XBP1S gene (5′-ATGCCAATGAACTCTTT CCCTTTT-3′) was cloned into a pSES-HUS vector (an adenoviral shuttle vector expressing siRNA) according to the manufacturer's instructions. Briefly, equimolar amounts of complementary sense and antisense strands were separately mixed, annealed and slowly cooled to 10°C in a 50-μl reaction buffer (100 mM NaCl and 50 mM HEPES, pH 7.4). The annealed oligonucleotides were inserted into the SfiI sites of pSES-HUS vector. All constructs were verified by nucleic acid sequencing; subsequent analysis was performed with BLAST software (National Institutes of Health, Bethesda, MD, USA).

Adenovirus XBP1S (Ad-XBP1S) siRNA, adenovirus encoding XBP1S and GEP were constructed, respectively, using methods described previously [46,59,60].

### Mice

All animal studies were performed in accordance with institutional guidelines and approval by the Institutional Animal Care and Use Committee of Chongqing Medical University. The GEP-knockout (GEP^−/−^) mice were bought from Jackson Laboratories (Bar Harbor, ME, USA), the generation and genotyping of GEP^−/−^ mice on basis of Jackson Laboratory's protocol were used for these experiments (http://jaxmice.jax.org/query/).

### Isolation and culture of mouse bone marrow stromal cells (BMSCs)

Mouse bone marrow was isolated by flushing the femurs and tibiae of 8- to 12-week-old female GEP^−/−^ knockout (GEP KO) mice with 0.6 ml of improved minimal essential medium (Sigma-Aldrich, St. Louis, MO, USA), supplemented with 20% foetal bovine serum (FBS), 100 units/ml penicillin, 100 μg/ml streptomycin (Invitrogen) and 2 mM glutamine (Invitrogen, Carlsbad, CA, USA), and then it was filtered through a cell strainer (Falcon, BD Biosciences, Franklin Lakes, NJ, USA). Cells were centrifuged for 10 min. at 260 × g, washed by the addition of fresh medium, centrifuged again, resuspended and plated out in improved minimal essential medium supplemented with 20% FBS, 100 units/ml penicillin, 100 μg/ml streptomycin and 2 mM glutamine at a density of 2 × 10^6^ cells/cm^2^ in 25-cm^2^ plastic culture dishes. The cells were incubated at 37°C in 5% CO_2_. After 72 hrs, non-adherent cells and debris were removed, and the adherent cells were cultured continuously. Cells were grown to confluence, washed with PBS and lifted by incubation with 0.25% trypsin, 2 mM ethylenediaminetetraacetic acid (Invitrogen) for 5 min. Non-detached cells were discarded, and the remaining cells were regarded as passage 1 of the BMSC culture. Confluent BMSCs were passaged and plated out at 1:2–1:3 dilutions. At passage 3, cells were transferred to DMEM (Invitrogen) supplemented with 10% FBS for differentiation studies.

### Cell culture

The micromass culture was performed as described previously [46]. Briefly, trypsinized C3H10T1/2 cells were resuspended in DMEM with 10% FBS at a concentration of 10^6^ cells/ml, and six drops of 100 μl of cells were placed in a 60-mm tissue culture dish (BD Biosciences). After a 2-hr incubation at 37°C, 1 ml of DMEM containing 10% FBS and BMP2 protein (300 ng/ml) was added. The medium was replaced approximately every 2–3 days. To test the effect of overexpression of XBP1S protein on chondrogenesis, C3H10T1/2 cells were infected with XBP1S expression adenovirus or control GFP adenovirus before micromass culture.

To test the effect of knocking down XBP1S on chondrogenesis, C3H10T1/2 cells were infected with Ad-XBP1S siRNA or control RFP adenovirus before micromass culture. Mouse chondrogenic ATDC5 cells were maintained in a medium consisting of a 1:1 mixture of DMEM and Ham's F-12 medium (Flow Laboratories, Irvine, UK) containing 5% FBS (Invitrogen), 10 mg/ml of human transferrin (Roche Applied Science, Penzberg, Germany) and 30 nM of sodium selenite (Sigma-Aldrich) at 37°C in a humidified atmosphere of 5% CO_2_ in air. The ATDC5 cells were seeded at a density of 3 × 10^5^ cells/well in 6-well cell culture plates (Corning Life Sciences, Edison, NJ, USA). The medium was replaced every other day. For adenovirus (Ad-XBP1S or Ad-GFP) infection and Ad-XBP1S siRNA and Ad-RFP infection, the same protocol as used with C3H10T1/2 cells was followed.

### Immunohistochemistry

Sections of post-coital day 12.5, 14.5, 15.5, 17.5 and 18.5 embryos and newborn mice were deparaffinized, rehydrated and placed in Tris buffer [10 mM Tris-HCl (pH 8.0), 150 mM NaCl]. Serum block was applied for 30 min. at room temperature before incubation of the primary antibody. Antimouse XBP1S (BioLegend, San Diego, CA, USA) was diluted 1:50, and sections were incubated at room temperature for 2 hrs. For detection, biotinylated secondary antibody and horseradish peroxidase (HRP)-streptavidin complex (Santa Cruz Biotechnology Inc., Santa Cruz, CA, USA) were used. Horseradish peroxidase substrate was used for visualization, and sections were then counterstained with Mayer's haematoxylin.

### Immunoblotting analysis

To examine the expression of XBP1S protein in the course of chondrogenesis, total cell extracts prepared from micromass cultures of ATDC5 cells in the presence of 300 ng/ml recombinant BMP2 protein were mixed with 5× sample buffer [312.5 mM Tris-HCl (pH 6.8), 5% β-mercaptoethanol, 10% SDS, 0.5% bromphenol blue, 50% glycerol]. Proteins were resolved on a 10% SDS-polyacrylamide gel and electroblotted onto a nitrocellulose membrane. After blocking in 10% non-fat dry milk in Tris buffer, saline Tween 20 [10 mM Tris-HCl (pH 8.0), 150 mM NaCl, 0.5% Tween 20], blots were incubated with mouse monoclonal anti-XBP1S antibody (diluted 1:500; BioLegend) for 1 hr. After washing, the respective secondary antibody [HRP-conjugated antimouse immunoglobulin (Sigma-Aldrich), both 1:1000 dilution] was added, and bound antibody was visualized using an enhanced chemiluminescence system (GE Healthcare, Little Chalfont, UK).

### Quantitative real-time PCR

To examine the effects of chondrogenesis by BMP2 and XBP1S, C3H10T1/2 or ATDC5 cells were plated at a density of 3 × 10^5^ cells/well in 6-well tissue culture plates. 300 ng/ml BMP2 or Ad-XBP1S (MOI 20) was then treated into these cells respectively. After day 3 or day 7, total RNAs were isolated using the RNeasy minikit (Qiagen, Hilden, Germany) and reverse transcribed into cDNA. Real-time PCR was performed with an ABI 7400 system using the TaqMan EZ RT-PCR kit according to the manufacturer's protocol. TaqMan primers and probes were derived from the commercially available TaqMan assay-on-demand gene expression products. We select GAPDH as the endogenous control for the real-time PCR relative quantification analysis. The following pair of oligonucleotides was used as internal controls: 3′-GTTTAGGCAAGTGTGGCTGGA-5′ and 3′-ACTGGAGTTGATGTACCAGATGT-5′for mouse GAPDH; 3′-GTGGTGGAAGAACTACAGTA-5′ and 3′-GTTCGAGTAAAGGACCAT CA-5′for human GAPDH. PCR cycling conditions were as follows: initial incubation step of 2 min. at 50°C, reverse transcription of 60 min. at 60°C and 94°C for 2 min., followed by 40 cycles of 15 sec. at 95°C for denaturation and 2 min. at 62°C for annealing and extension.

In the case of collagen II, collagen X, RUNX2 real-time PCR was run using the SYBR Green PCR kit, and the following primers were used: sense (3′-AACGAGAACGACGAGGTGGT-5′) and antisense (3′-AAAGGAGGCAGATGACAGGTGAC-5′) for collagen II; sense (3′-TAC CACGTGCATGTGAAAGG-5′) and antisense (3′-GGAGCCACTAGGAATCCTGAG-5′) for collagen X; sense (3′-TCAAACGCCTCTTCAGCGCAGTG-5′) and antisense (3′-GGCT GGTGCTCGGATCCCAAAAGA-5′) for RUNX2.

### Reporter gene assays

Micromass culture of ATDC5 cells were plated at a density of 3 × 10^5^ cells/well in 6-well tissue culture plates and transfected with XBP1-specific reporter plasmids (pGL3-XBP1-luc) and pCMV-gal (an internal control for transfection efficiency). Forty-eight hours after transfection, cells were harvested, and luciferase and β-galactosidase activity was measured using the Bioscan Mini-Lum luminometer. Relative transcriptional activity was expressed as a ratio of luciferase reporter gene activity from the experimental vector to that from the internal control vector. The cultures were processed and analysed as described above.

### Chromatin immunoprecipitation

Micromass culture of ATDC5 cells treated with BMP2 was fixed by 1% formaldehyde for 10 min. before cell lysis. Cell lysates were subsequently sonicated, followed by centrifugation. The input (1% of the supernatant) was used in PCR as a positive control. The supernatant was then pre-cleared using protein A-agarose/salmon sperm DNA for 30 min. at 4°C. After centrifugation, the supernatant was then used for immunoprecipitation using an anti-Smad4 antibody or the control IgG antibody and incubated overnight at 4°C. The protein-DNA complex was subsequently incubated with protein A-agarose/salmon sperm DNA for 1 hr at 4°C. The immune complex was collected by centrifugation and then washed five times with the following for 5 min. each: once with low salt immune complex wash buffer, once with high salt immune complex wash buffer, once with LiCl salt immune complex wash buffer and twice with TE buffer. The histone-DNA complex was eluted from the antibody using elution buffer (1% SDS, 0.1 M NaHCO_3_), and 5 M NaCl was added to reverse the histone-DNA cross-link by heating for 4 hrs at 65°C. The DNA was then extracted with phenol/chloroform and precipitated with ethanol in the presence of glycogen (20 mg) as a carrier. The precipitate was used as a template for PCR amplification. For PCR of the XBP1S minimal promoter region using the chromatin-immunoprecipitated DNA, one-tenth of the DNA was PCR amplified using forward primer, 5′-CAATGGACGCCGAGCTCG-3′; and reverse primer, 5′-CATAGCTCCAGACTAC GC-3′. Thirty-five cycles of PCR at 94°C for 30 sec., 55°C for 30 sec. and 72°C for 30 sec. were performed. PCR products were analysed by 1% agarose gel.

### Culture of foetal mouse bone explants

Foetal mouse metatarsals were dissected from foetal GEP null mice (GEP^−/−^, 15-day-old embryos) and cultured in DMEM (Gibco, Carlsbad, CA, USA) containing 1% heat-inactivated foetal calf serum (Invitrogen) and 100 U penicillin-streptomycin per milliliter in the absence or presence of various stimuli for 5 days, as indicated in Figures4, 8 and 9.

For alizarin red and alcian blue staining (staining for bone and cartilage), the explants were placed in 4% paraformaldehyde in phosphate-buffered saline for overnight fixation. Subsequently, explants were placed in staining solution (0.05% alizarin red, 0.015% alcian blue, 5% acetic acid in 70% ethanol) for 45–60 min. Digital images of stained bones were analysed. For safranin O-fast green staining, explants were fixed in 96% alcohol and processed for paraffin embedding. Sections were stained with 0.1% safranin O (orange stain) to evaluate cartilage matrices and with 0.03% fast green to evaluate morphological features as previously described [57].

### Immunocytostaining of GEP induced by XBP1S

To reveal the induction of GEP by XBP1S, the C28I2 cells were transiently transfected with the pcDNA3.1(-) expression vector containing cDNA encoding XBP1S. Then, detection of the expression of GEP in the control and XBP1S-treated C28I2 cells was performed, the cells were washed, fixed with 100% methanol in the freezer compartment for 5 min., washed twice in 4°C phosphate-buffered saline for 5 min. and then incubated with 30% goat serum in phosphate-buffered saline for 30 min.; the cells were incubated with primary antibodies (*i.e*. mouse monoclonal anti-GEP antibodies) at room temperature for 1 hr. After being washed with phosphate-buffered saline, the coverslips were incubated with secondary antibodies (*i.e*. goat antimouse IgG conjugated with rhodamine; Santa Cruz Biotechnology; diluted 1:100) and goat anti-rabbit IgG conjugated with fluorescein isothiocyanate (Santa Cruz Biotechnology; diluted 1:100) for 1 hr. The specimens were observed under a fluorescence microscope with appropriate optical filters. Microscopic images were captured by using the Image-Pro programme (Media Cybernetics, Sarasota, FL, USA) and an Olympus microscope. Images were arranged using Adobe Photoshop.

### Statistical analysis

The statistical analysis was performed with SPSS 10.0.1 software (Chicago, IL, USA) for Windows. Data were expressed as mean ± SD from at least three independent experiments. Data for multiple variable comparisons were analysed by one-way anova. *P* < 0.05 was deemed statistically significant.

## Results

### Differential expression of XBP1S in the chondrogenesis of a micromass culture of ATDC5 and C3H10T1/2 cells

It is reported that ER stress signal molecules were associated with chondrogenesis [33–35]. In this study, we sought to determine whether XBP1S, a vital transcription factor in ER stress, participates in cartilage development. We first studied XBP1S expression profiles during chondrocyte differentiation using the ATDC5 cell line and C3H10T1/2 cell line [36–38].

**Figure 1:**
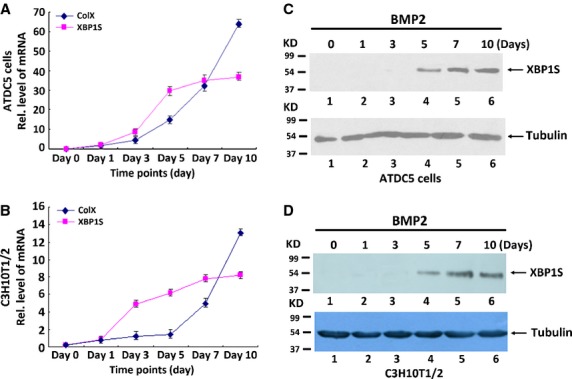
Expression of XBP1S in the course of chondrogenesis *in vitro*. (A and B) Expression of XBP1S and Collagen X was examined in the course of chondrogenesis of a micromass culture of ATDC5 cells (A) and C3H10T1/2 cells (B). Micromass cultures of ATDC5 cells or C3H10T1/2 cells were stimulated with BMP2 protein for various time-points, respectively, as indicated, and the mRNA levels of XBP1S and ColX and GAPDH (serving as an internal control) were examined by real-time PCR. Rel. level, relative level. (C and D) Differential expression of XBP1S protein was examined by western blotting assay. After incubation of micromass cultures of ATDC5 cells (C) or C3H10T1/2 cells (D) with 300 ng/ml BMP2 for the various times indicated, the cells were lysed, and the levels of XBP1S and tubulin (serving as an internal control) were detected by using immunoblotting assay.

It differentiates specifically to the cartilage lineage at high yields when inoculated under high-cell-density micromass cultures as well as when exposed to chondroinductive factors such as a well-documented growth factor BMP2 [5,39]. Therefore, both ATDC5 and C3H10T1/2 cells have the potential to become chondrocytes, making them a valuable *in vitro* correlate for studying the mechanisms of chondrogenesis. To obtain XBP1S expression profiles during chondrocyte differentiation, micromass cultures of ATDC5 and C3H10T1/2 cells were incubated in the presence of 300 ng/ml of recombinant BMP2 for induction of chondrocyte differentiation. Cells were harvested at various time-points and then followed by real-time PCR for measures of XBP1S and collagen X (a specific marker for hypertrophic chondrocytes). As shown in Figure1A and B, the level of XBP1S mRNA was relatively low until day 5; when it had doubled, and thereafter remained at high levels during the differential stage, representing terminal differentiation marked by the increase in collagen X expression. In addition, similar results were also observed in the course of chondrogenesis of C3H10T1/2 cells. It is noteworthy, that the peak level of XBP1S was 2 days earlier than that of collagen X, suggesting that XBP1S may regulate collagen X expression.

We next examined the level of XBP1S protein. Micromass culture of ATDC5 and C3H10T1/2 cells were harvested at various time-points, respectively, followed by Western blotting (Fig.1C and D). XBP1S protein was markedly elevated at day 5 and thereafter, remained at high levels.

### XBP1S expression patterns in chondrocytes during both embryonic and post-natal development stages

**Figure 2:**
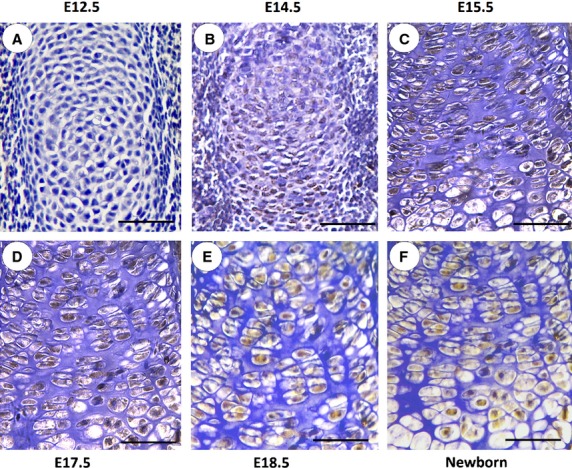
Immunohistochemistry of XBP1S in tibial growth plate chondrocytes *in vivo*. Temporal and spatial expression of XBP1S during chondrogenesis in tibial growth plates of post-coital day 12.5 mouse embryo (E12.5; A); post-coital day 14.5 mouse embryo (E14.5; B); post-coital day 15.5 mouse embryo (E15.5; C); post-coital day 17.5 mouse embryo (E17.5; D); post-coital day 18.5 mouse embryo (E18.5; E); and newborn (F) is shown. Microphotographs are shown of sections stained with anti-XBP1S antibody (brown) and counterstained with haematoxylin (blue). Immunostaining reveals positive nuclear staining in the entire chondrogenic developmental stages in both proliferating and hypertrophic zones. The scale bars represent 100 μm.

Next, we characterized the temporal and spatial expression pattern of XBP1S during skeletal development using an immunostaining assay at multiple time-points, including embryonic day 12.5 (E12.5; onset of chondrogenesis that begins with the proliferation and subsequent condensation of mesenchymal cells), E14.5 (right after cartilage formation but before endochondral bone formation) and E15.5 (onset of skeletal growth), as well as E17.5, E18.5 and newborn. As revealed in Figure2, XBP1S is detected at E14.5, and its level is increased in the centre of the condensation and around it at E15.5. It demonstrates prominent expression in pre-hypertrophic chondrocytes at E15.5 and E17.5, E18.5 and in newborn mice. A high level of XBP1S throughout the whole growth plate is observed at E17.5, E18.5 and newborn mice, suggesting that the expression profile of XBP1S is closely linked to the entire chondrogenic period.

### XBP1S stimulates chondrogenesis *in vitro* and endochondral bone formation *ex vivo*

Prominent expression of XBP1S in chondrocytes prompted us to determine whether XBP1S was able to induce chondrocyte differentiation. We next sought to determine the role of XBP1S and BMP2 (300 ng/ml) during chondrogenesis in micromass cultures of pre-chondrogenic ATDC5 cells and BMSC cells, which are capable of differentiation into various lineages, including chondrocytes [36–38].

**Figure 3:**
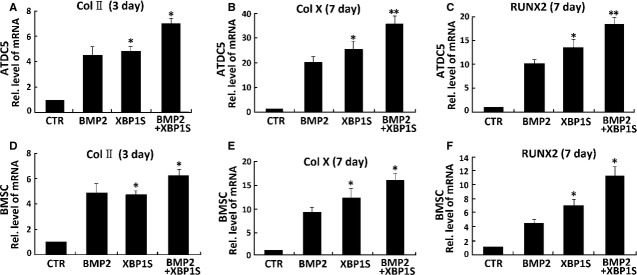
XBP1S stimulates chondrogenesis *in vitro*. (A) Comparisons of XBP1S and BMP2 in stimulations of chondrogenesis of ATDC5 cells and murine bone marrow stromal cells (BMSCs; B). Micromass cultures of ATDC5 and BMSC cells were incubated in the absence (CTR) or presence of either 300 ng/ml BMP2, Ad-XBP1S (MOI 20) and Ad-XBP1S (MOI 20)+BMP2 for 3 or 7 days, and the mRNA levels of Col II (A and D), Col X (B and E), RUNX2 (C and F) were determined using real-time PCR. The units are arbitrary, and the normalized values were calibrated against control (CTR), here given the value of 1. The asterisk (*) indicates significant increase or decrease from control (*P < 0.05).

In brief, the high-density culture system was incubated in the absence (CTR) or presence of Ad-XBP1S or 300 ng/ml BMP2 (serving as a positive control) for 3 or 7 days. Chondrogenesis was monitored by analysing the expressions of marker genes specific for chondrocytes (Fig.3). ATDC5 or BMSC cells were treated with BMP2, adenovirus encoding XBP1S (Ad-XBP1S), Ad-XBP1S+BMP2 and control GFP (Ad-GFP), respectively, then, RNA was extracted every other day for real-time PCR.

As revealed in Figure3A–F, chondrocyte differentiation was monitored by examining the expression of collagen II, collagen X and RUNX2, three marker genes widely used for chondrocyte maturation and hypertrophy [4,5]. As for BMP2, XBP1S markedly induced the expression of collagen II, collagen X and RUNX2. Besides, clearly enhanced expressions of collagen II, collagen X and RUNX2 in Ad-XBP1S+BMP2-treated cells were observed compared with those in BMP2-treated or Ad-XBP1S-treated cells, suggesting that XBP1S can enhance BMP2-induced chondrogenesis, thus, XBP1S is a positive mediator for chondrocyte differentiation and hypertrophy.

**Figure 4:**
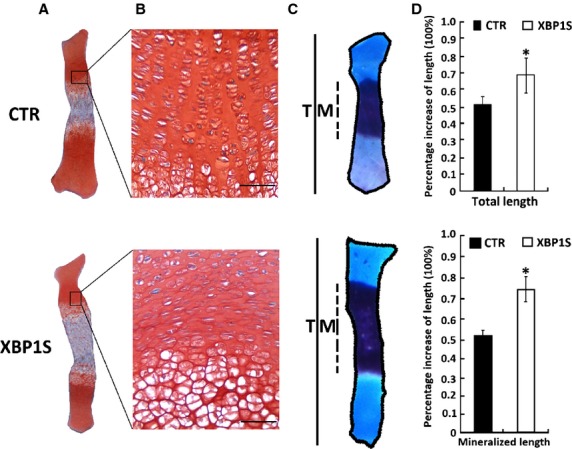
XBP1S stimulates chondrocyte hypertrophy, mineralization and endochondral bone growth. (A and B) Safranin O/Fast Green staining of metatarsal bones. Metatarsals from 15-day-old mouse embryos were cultured in the absence of (CTR) or presence of Ad-XBP1S (MOI 20) for 5 days and stained with Safranin O/Fast Green, shown in low-power (A) and high-power (B) microphotographs. (C) Alizarin red/Alcian blue staining of metatarsals. Explants were fixed and processed for staining. A representative photograph of an explanted metatarsal is presented. (D) Percentage changes in total (T) and mineralization (M) length of metatarsal bones. Percentage changes in bone length were calculated as (length at d5 − length at d0)/length at d0. *P < 0.05 *versus* control; scale bar = 100 μm.

The effect of XBP1S on endochondral bone formation was then studied in an *ex vivo* model of 15-day-old foetal mouse metatarsal bones. At the time of explantation, these explants consisted of undifferentiated cartilage. In a 5-day culture period of Ad-XBP1S (MOI 20), these explants underwent all sequential stages of endochondral bone formation. As shown in Figure4, XBP1S significantly stimulated chondrocyte hypertrophy, mineralization and bone length.

### XBP1S is a BMP2-inducible transcription factor and required for BMP2-mediated chondrocyte differentiation

**Figure 5:**
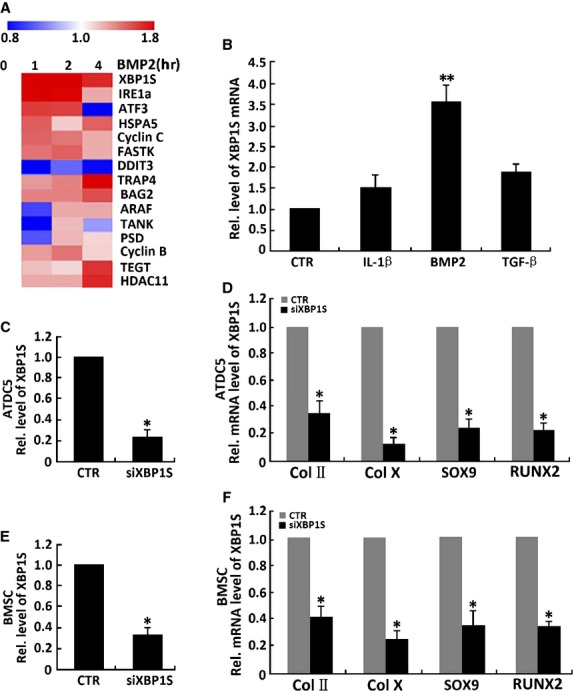
XBP1S is a downstream molecule of BMP2 and is required for BMP2 stimulation of chondrogenesis. (A) Genome-wide DNA chip analysis for isolating BMP2-responsive genes. Total RNA was isolated from human C28I2 chondrocytes treated with 300 ng/ml BMP2 for various time-points, as indicated, and analysed by microarray analysis. Several up-regulated genes after BMP2 treatment were determined by hierarchical clustering. (B) Effects of cytokines on XBP1S mRNA in chondrocytes by real-time PCR. Expression of XBP1S mRNA was normalized against GAPDH (serving as an internal control). **P < 0.01. (C) siRNA against XBP1S mRNA efficiently inhibited expression of endogenous XBP1S in ATDC5 cells. siXBP1S reduces 81% of endogenous XBP1S mRNA in ATDC5 cells. Cells were infected with either siXBP1S adenovirus (MOI 20) or control adenovirus (CTR), and total RNA was collected for real-time PCR. Expression of XBP1S was normalized against the GAPDH endogenous control. The normalized values were then calibrated against the control value, here set as 1. *P < 0.05. (D) Suppression of XBP1S by siRNAs inhibits BMP2-induced chondrogenesis in ATDC5 cells. Micromass cultures of C3H10T1/2 cells infected with either control adenovirus or siXBP1S adenovirus (MOI 20) were used to test whether BMP2 (300 ng/ml)-induced chondrogenesis is XBP1S dependent. Expressions of marker genes, as indicated, were determined by real-time PCR. (E) siRNA against XBP1S mRNA efficiently inhibited expression of endogenous XBP1S in bone marrow stromal cells (BMSC). siXBP1S adenovirus reduces 72% of endogenous XBP1S mRNA in BMSC cells. The method is the same with C. (F) siXBP1S adenovirus inhibits BMP2-induced chondrogenesis in BMSC cells. The method is the same with D.

To identify BMP2 downstream molecules, we performed genome-wide DNA chip analysis (Fig.5A). Total RNA was isolated from human C28I2 chondrocytes treated with 300 ng/ml BMP2 at various time-points and analysed by microarray analysis (Shanghai Kangcheng Biotechnology, Shanghai, China). Approximately 50 genes were up-regulated (twofold) by BMP2, as determined by hierarchical clustering, and some of the BMP2-inducible genes (Fig.5A), including XBP1S, IRE1α, ATF3, HSPA5, DDIT3 are also known to be activated by ER stress [41–45]. We focused on XBP1S, because BMP2 mediates ER stress, and IRE1α-XBP1 pathway is an important UPR signal pathway.

To testify the result of genome-wide DNA chip analysis, we tested a few cytokines known to be important for chondrogenesis in the primary human chondrocytes. Our results showed that XBP1S mRNA is up-regulated threefold by BMP2 and 1.5-fold by TGF-β. IL-1β had no apparent effects on XBP1S expression (Fig.5B).

Next, we tested whether XBP1S was required for BMP2-mediated chondrogenesis using the siRNA approach. As shown in Figure5C and E, infection with siXBP1S adenovirus resulted in 81% and 72% reduction in XBP1S mRNA in ATDC5 cells and BMSC cells respectively. Micromass cultures of ATDC5 cells or BMSC cells infected with siXBP1S adenovirus or control adenovirus (CTR) were treated with BMP2 for various time-points. As shown in Figure5D and F, our real-time PCR assay showed that reductions of the endogenous XBP1S by siXBP1S adenovirus sharply decrease chondrogenic responses induced by BMP2: 77% down of Sox9, 62% down of collagen II, 83% down of collagen X and 75% down of RUNX2 compared with the control group responses in ATDC5 cells (Fig.5D); 67% down of Sox9, 60% down of collagen II, 78% down of collagen X and 65% down of RUNX2 compared with the control group responses in BMSC cells (Fig.5F). These results support the concept that XBP1S is a key downstream molecule of BMP2 during chondrocyte development.

### BMP2 and Smads activate XBP1S-specific reporter genes

**Figure 6:**
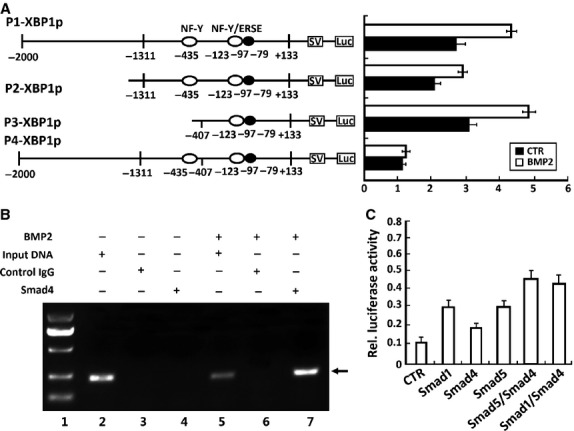
BMP2 and its mediators, Smads, activate the XBP1S-specific reporter genes. (A) BMP2 activates XBP1S-specific reporter genes in ATDC5 cells. Indicated segments from the 5′-flanking region of the XBP1S gene were linked to a simian virus 40 promoter and a DNA segment encoding luciferase. Numbers indicate distances in nucleotides from first nucleotide of intron 1. Indicated reporter gene and a pSVgal internal control plasmid were transfected into ATDC5 cells in the presence or absence of 300 ng/ml BMP2 for 48 hrs, and β-galactosidase and luciferase activities were determined. Luciferase activities were normalized toβ-galactosidase activities. (B) BMP2-activated Smad4 binds to the XBP1S promoter (ChIP). Micromass culture of ATDC5 cells treated with or without 300 ng/ml BMP2 for 12 hrs was cross-linked by formaldehyde treatment and lysed. Cell lysates were subjected to immunoprecipitation with control IgG or anti-Smad4. Purified DNA from the cell lysate (input DNA serves as a positive control) and DNA recovered from immunoprecipitation were amplified by PCR using specific primers for XBP1S minimal promoter. (C) BMP2 downstream transcription factors (Smads) activate the XBP1S-specific reporter genes. XBP1S-specific reporter construct −407XBP1Sluc was transfected into ATDC5 cells together with the indicated Smad expression plasmids (*i.e*. Smad1, Smad4 and Smad5), as well as a pSVgal internal control plasmid. At 48 hrs after transfection, cultures were harvested and processed as described in A.

To elucidate the molecular mechanism by which BMP2 activates XBP1S expression, firstly, four XBP1S-specific reporter gene plasmids, −2000→+133XBP1Sluc [labelled p1], −1311→+133 XBP1Sluc [p2], −407→+133XBP1Sluc [p3] and −2000→−407 XBP1Sluc [p4], were generated in which segments of the XBP1S promoter, with or without the NF-Y or NF-Y/ERSE binding site, were inserted upstream of the luciferase coding region of the pGL3 basic vector (Fig.6). On the other hand, deletion of the region from −407 to +133 leads to the complete loss of the reporter activity, indicating that this region is probably the basic promoter of the XBP1S gene. The core sequence of XBP1S promoter is found from −407 to +133 bp. Applications of BMP2 were able to activate all XBP1S promoter constructs containing the region between −407 and +133. Furthermore, this basic promoter region directly responded to BMP2 (Fig.6A).

Because BMP2 activates the cellular signalling through Smads, we then tested interaction of Smad4, a coregulatory Smad that binds to Smad1 or Smad5 for transducing BMP2 signalling, with XBP1S promoter regions (in particular, the region of −407 and +133) *in vitro* using the ChIP assay. As shown in Figure6B, we observed a clear PCR product using DNA isolated from immunoprecipitated complexes with anti-Smad4 antibodies from BMP2-treated cells, but not from BMP2-untreated cells, suggesting that the Smad4 is recruited into this XBP1S promoter region after exposure to BMP2.

Next, we determined whether Smad transcription factors could directly activate the XBP1S at the transcription level. Cotransfection of the XBP1S luciferase plasmid (−407XBP1Sluc) with an expression plasmid encoding either Smad1, Smad4 and Smad5 (cDNA constructs kindly provided by Dr. Chuanju Liu, Department of Orthopaedic Surgery and Department of Cell Biology, New York University School of Medicine), or a combination of either Smad1/Smad4 or Smad5/Smad4, markedly increased the expression of the XBP1S reporter gene. Both of the combinations of Smad1/Smad4 and combinations of Smad4/Smad5 gave the higher value than the others (Fig.6C). The above data support the notion that BMP2 controls XBP1S expression through Smad signalling.

### XBP1S induces GEP expressions in C3H10T1/2 and ATDC5 cells

**Figure 7:**
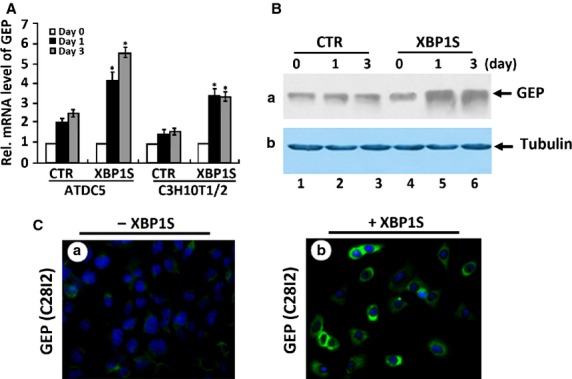
XBP1S increases granulin-epithelin precursor (GEP) expression in chondrogenesis. (A) XBP1S induces the expression of GEP mRNA, assayed by real-time PCR. 10T1/2 and ATDC5 cells pre-treated with recombinant 300 ng/ml of BMP2 for 1 week were cultured without Ad-XBP1S (CTR) or with Ad-XBP1S (MOI 20) for various time periods, as indicated. The normalized values against GAPDH were calibrated against controls (day 0), given the value of 1. Asterisk indicates a significant difference from the control at corresponding time-points (*P* < 0.05). (B) XBP1S induces the expression of GEP protein, assayed by Western blotting. ATDC5 cells pre-treated with recombinant 300 ng/ml of BMP2 for 1 week were cultured without Ad-XBP1S (CTR) or with Ad-XBP1S (MOI 20) for various time periods, as indicated. The cell lysates were detected with either anti-GEP or anti-tubulin (serving as an internal control) antibodies. (C) XBP1S increases the level of GEP protein, assayed by immunofluorescent cell staining. C28I2 cells treated with or without pcDNA3.1(-)-XBP1S for 24 hrs were stained with anti-GEP antibodies (green). The nuclei were stained with 4′,6-diamidino-2-phenylindole (DAPI).

We have found that XBP1S is expressed throughout the whole growth plate at E17.5, E18.5 and newborn mice (Fig.2) and positively regulates chondrocyte development (Figs3 and 4). We previously reported that GEP is a key downstream molecule of BMP2, and it is required for BMP2-mediated chondrocyte differentiation. We next used C3H10T1/2 cells and chondroprogenitor ATDC5 cells to examine the relationship between GEP and XBP1S. Micromass cultures of both C3H10T1/2 and ATDC5 cells pre-treated with 300 ng/ml of BMP2 for 1 week were cultured with or without Ad-XBP1S for various time-points, and the level of GEP mRNA was measured by using real-time PCR (Fig.7A).

Granulin-epithelin precursor mRNA was increased to 2.0-fold at day 1 and to 2.3-fold by day 3 in the XBP1S-untreated control ATDC5 cells. XBP1S markedly enhanced the level of GEP mRNA to 4.2-fold at day 1 and to 5.7-fold by day 3 in the XBP1S-treated ATDC5 cells. In the case of C3H10T1/2 cells, GEP mRNA was slightly increased to 1.3-fold at day 1 and to 1.5-fold by day 3 in the XBP1S-untreated cells; And XBP1S significantly induced GEP to 3.6-fold at day 1 and to 3.5-fold by day 3 in the XBP1S-treated C3H10T1/2 cells. GEP mRNA in the XBP1S-treated cells was approximately twofold higher than the mRNA in the control at the same time-point. In addition, induction of the GEP protein level by XBP1S was also visualized by both immunofluorescent cell staining in C28I2 chondrocytes (Fig.7C) and immunoblotting in ATDC5 cells (Fig.7B). Taken together, these findings demonstrate that GEP is a XBP1S-inducible gene in the process of chondrogenesis.

### XBP1S activates chondrogenesis and endochondral bone formation through GEP

**Figure 8:**
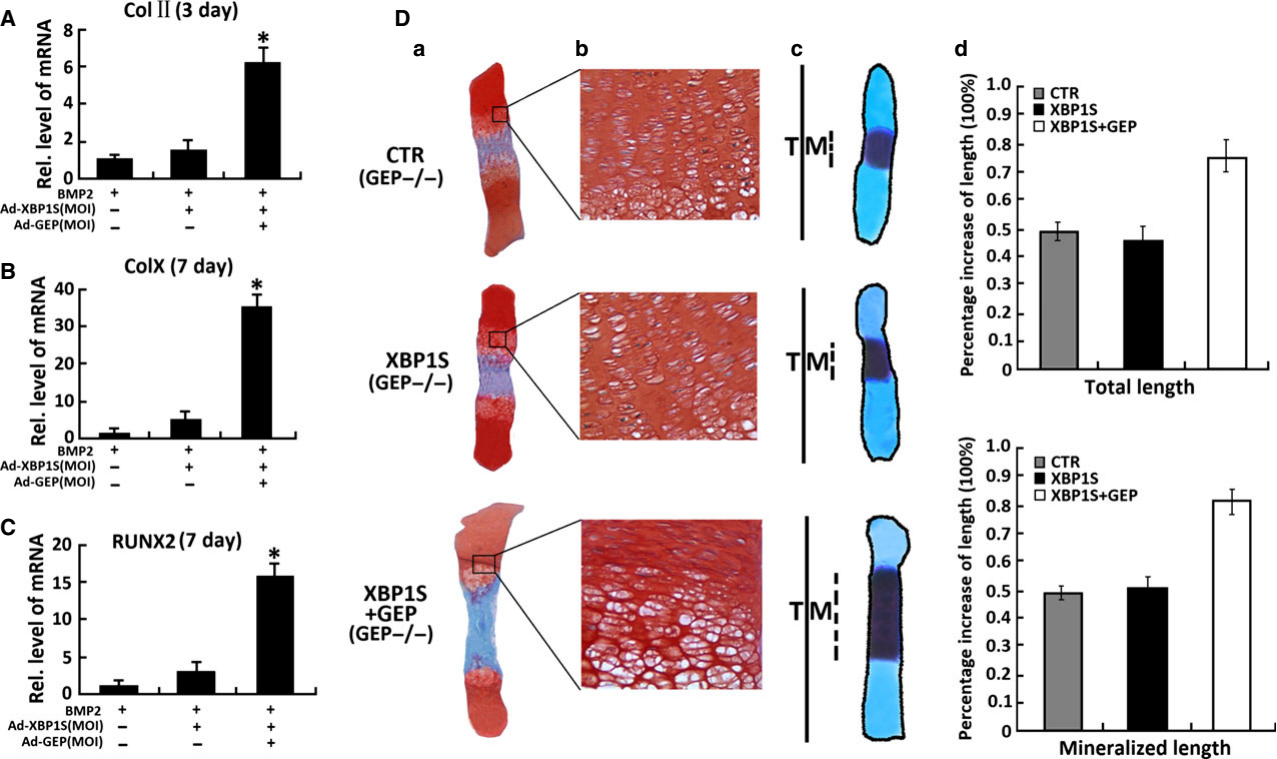
Granulin-epithelin precursor (GEP) is required for the XBP1S-induced chondrocyte differentiation and endochondral bone formation. (A) XBP1S stimulates chondrocyte differentiation through GEP. Micromass culture of GEP^−/−^ bone marrow stromal cells (BMSC) infected with or without Ad-XBP1S (MOI 20) and Ad-GEP (MOI 20) for 3 days was used to test whether BMP2 (300 ng/ml)-induced chondrogenesis is GEP dependent. Expressions of marker genes Col II, as indicated, were determined by real-time PCR; (B) Micromass culture of GEP^−/−^ BMSC cells infected with or without Ad-XBP1S (MOI 20) and Ad-GEP (MOI 20) for 7 days was used to test whether BMP2 (300 ng/ml)-induced chondrogenesis is GEP dependent. Expressions of marker genes Col X, as indicated, were determined by real-time PCR; (C) Micromass culture of GEP^−/−^ BMSC cells infected with or without Ad-XBP1S (MOI 20) and Ad-GEP (MOI 20) for 7 days was used to test whether BMP2 (300 ng/ml)-induced chondrogenesis is GEP dependent. Expressions of marker genes RUNX2, as indicated, were determined by real-time PCR; (D) XBP1S increased BMP2-induced chondrocyte hypertrophy, mineralization and bone length through GEP. (a and b) Safranin O-fast green staining of metatarsals. Metatarsals were explanted from 15-day-old GEP null mouse embryos and cultured in the absence (CTR) or presence of Ad-XBP1S (MOI 20), Ad-GEP (MOI 20). The explants were cultured for 5 days, and safranin O-fast green staining was observed by using low-power (a) or high-power (b) microphotography. (c) Alizarin red S and alcian blue staining of metatarsals. Metatarsals were cultured as described above and processed for alizarin red S and alcian blue staining; a representative photograph is presented. (d) Per cent increase in total and mineralization length of metatarsal bones. Metatarsals were cultured as described above, total or mineralization length was determined, and the per cent increase was calculated (per cent increase = [length at day 5 − length at day 0]/length at day 0). Asterisk indicates a significant difference from the control (*P* < 0.05).

We next investigated whether endogenous GEP is required for XBP1S-induced chondrocyte development. Firstly, we isolated GEP null mice (GEP^−/−^) BMSC cells, then performed micromass culture of GEP^−/−^ BMSC cells. As shown in Figure8A–C, Realtime PCR results showed that Ad-XBP1S cannot promote BMP2-induced Col II (Fig.8A), Col X (Fig.8B) and RUNX2 (Fig.8C) expression in GEP^−/−^ BMSC cells, however, after infection with Ad-GEP, Ad-XBP1S can increase the expression of Col II (Fig.8A), Col X (Fig.8B) and RUNX2 (Fig.8C) induced by BMP2 in GEP^−/−^ BMSC cells. It was indicated that Ad-XBP1S activates BMP2-induced chongenesis through GEP growth factor.

In addition, the dependence on GEP of XBP1S-mediated endochondral bone formation was revealed by using cultures of 15-day-old foetal GEP null mice metatarsal bones (Fig.8D). In line with a previous report [46], XBP1S potently enhanced chondrocyte hypertrophy; and the effect of XBP1S-induced chondrogenesis and endochondral bone formation was largely abolished in GEP^−/−^ BMSC cells. These results indicated that XBP1S-mediated chondrocyte differentiation and endochondral bone growth depends, at least in part, on GEP. We next sought to determine whether XBP1S recovered the valid stimulating in growth plates of GEP^−/−^ embryos rescued by GEP with safranin O-fast green staining. As shown in Figure8D, disorganized GEP null growth plates, including reductive chondrocyte hypertrophy, cannot be changed by Ad-XBP1S, however, it can be largely corrected in the presence of Ad-XBP1S+Ad-GEP. XBP1S recovered the potent stimulating effect of chondrocyte differentiation, mineralization and endochondral bone growth in GEP null growth plates rescued by GEP.

Taken together, endogenous GEP is required for XBP1S-stimulated chondrocyte hypertrophy, mineralization and endochondral bone formation.

### XBP1S enhances the chondroinductive activity of GEP

Then, we examined whether XBP1S-mediated augment of chondrocyte hypertrophy and endochondral bone growth is exerted by activating GEP's chondroinductive activity. We previously reported that GEP is a novel growth factor increasing chondrocyte differentiation and endochondral bone formation, and cartilage repair [21,22]. For this purpose, we first examined whether XBP1S was able to increase GEP-stimulated chondrocyte hypertrophy using chondroprogenitor ATDC5 cells.

**Figure 9:**
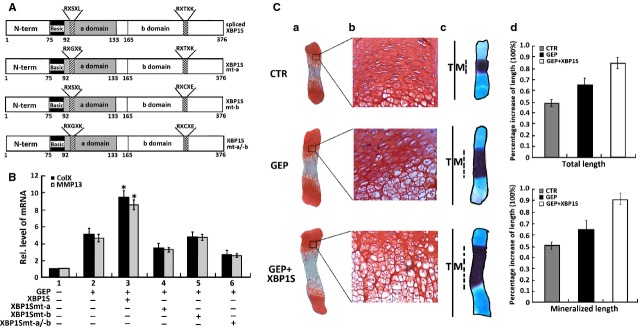
XBP1S activates the chondroinductive action of granulin-epithelin precursor (GEP). (A) Schematic structures of wild-type and mutant XBP1S proteins. The N-terminal domain (N-term), one DNA binding domain (type a domain) and one transactivating domain (type b domain) are indicated. RXSXL and RXTXK are the amino acid sequences in the DNA binding domain and the transactivating domain. In the XBP1Smt-a mutant, the RXSXL in the a domain was substituted by RXGXK, in the XBP1Smt-b mutant, the RXTXK in the b domain was substituted by RXCXE, and in the XBP1Smt-a/b double mutant, the RXSXL and RXTXK motifs in both a and b domains were replaced by RXGXK and RXCXE. (B) XBP1S improves GEP-induced Col X and MMP-13 expression, while XBP1S mutants failed to enhance the GEP-induced Col X and MMP-13 expression, assayed by real-time PCR. ATDC5 cells pre-treated with BMP2 for 5 days were cultured in the absence or presence of Ad-GEP (MOI20), Ad-XBP1S (MOI20)+Ad-GEP, Ad-XBP1Smt-a (MOI20)+Ad-GEP, Ad-XBP1Smt-b (MOI 20)+Ad-GEP, Ad-XBP1Smt-a/-b (MOI 20) + Ad-GEP as indicated for an additional 3 days, and Col X and MMP-13 expression was measured by real-time PCR. The units are arbitrary, and the normalized values were calibrated against controls, here given the value of 1. Asterisks indicate a significant difference from the control (*P* < 0.05). (C) XBP1S enhances GEP-stimulated endochondral ossification. (a and b) Safranin O-fast green staining of metatarsals. Metatarsals were explanted from 15-day-old mouse embryos and cultured in the absence (CTR) or presence of Ad-GEP (MOI 20) with or without Ad-XBP1S. After 5 days of culture, safranin O-fast green staining was observed by using low-power (a) or high-power (b) microphotography. (c) Alizarin red S and alcian blue staining of metatarsals. The explants were fixed and processed for alizarin red S and alcian blue staining; a representative photograph is presented. (d) Per cent increase in total and mineralization length of metatarsal bones. Metatarsals were cultured as described above, the total or mineralization length was determined and the per cent increase was calculated (per cent increase = [length at day 5 − length at day 0]/length at day 0). Asterisks indicate a significant difference from the control (*P* < 0.05).

As noted in Figure9A, XBP1S contains a DNA binding domain (a domain) and a transactivating domain (b domain; Fig.9A, top scheme). We generated XBP1S derivatives with mutations in the DNA binding domain (XBP1Smt-a), the transactivating domain (XBP1Smt-b) or both domains (XBP1S mt-a/b; Fig.9A, three lower schemes).

Then, ATDC5 cells pre-treated with BMP2 for 1 week were cultured without or with Ad-GEP (MOI 20), Ad-XBP1S (MOI 20) or its point mutation Ad-XBP1Smt-a, Ad-XBP1Smt-b, Ad-XBP1Smt-a/-b or various combinations as indicated in Figure9B. Realtime PCR results showed that GEP-stimulated Col X and MMP-13 expressions were remarkably increased by the addition of Ad-XBP1S. In BMP2-induced ATDC5 cells infected with Ad-XBP1S, GEP-induced Col X and MMP-13 expression were increased ∽2.2-fold, whereas XBP1S in which either of the DNA binding domain and the transactivating domain were mutated, failed to enhance the GEP-induced Col X and MMP-13 expression (Figure9B). This augment of GEP action depends on XBP1S activity, as the XBP1S point mutation failed to do so. These results indicate that both the DNA binding domain and the transactivating domain of XBP1S are needed for stimulating GEP-dependent chondrogenesis.

We next determined whether XBP1S was also able to improve the GEP activity in regulating endochondral bone growth. As expected, GEP growth factor stimulated chondrocyte maturation, mineralization and bone growth; and GEP-mediated endochondral bone growth was clearly increased by the addition of Ad-XBP1S (Fig.9C). These observations, together with the finding that GEP is required for the XBP1S-induced chondrocyte differentiation and endochondral bone formation, suggested that XBP1S positively regulates chondrocyte hypertrophy and endochondral bone growth through stimulating with GEP and activating its chondrogenic activity.

## Discussion

Growth and development of endochondral bones is regulated through the well-orchestrated proliferation and differentiation of growth plate chondrocytes. Chondrogenesis is a process that is important for cartilage remodelling both during embryogenesis and in adult life [47,48]. The IRE1/XBP1 branch of the UPR is known to be essential for normal development. XBP1S is required for the terminal differentiation of B cells, hepatocytes and pancreatic β cells. It is also important for myeloma cells to survive hypoxic stress [49,50]. Many studies have shown that factors influencing cell fate and/or differentiation are activated in ER stress [51,52], but how such changes impact differentiation programmes in chondrocytes is poorly understood. Therefore, to test a link between the IRE1/XBP1 branch of the UPR and chondrocyte differentiation, we focused on the role of XBP1S in chondrogenesis as well as the molecular mechanism involved.

Our results showed that XBP1S protein was highly induced in the course of BMP2-stimulated chondrogenesis *in vitro* (Fig.1) and also demonstrated prominent expression in the entire growth plate chondrocyte population *in vivo* (Fig.2). Real-time PCR for measurements of XBP1S showed that the level of XBP1S mRNA was relatively low until day 5, and at day 7, it tripled and thereafter remained at high levels during the late differential stage (Fig.1A and B). The different expression between the protein and mRNA of XBP1S during chondrogenesis suggests that post-transcription regulations, such as mRNA stability, translation and protein degradation, might be also important in the control of XBP1S expression during chondrogenesis. The *in vitro*, *ex vivo* and *in vivo* studies support the concept that XBP1S is a potent stimulator of chondrocyte differentiation, mineralization and endochondral bone growth (Figs3 and 4).

Saito *et al*. [35] reported that BMP2 induced ER stress in osteoblasts, and ER stress-inducing agents activate the IRE1α/β proteins. IRE1α, a kind of ER type I transmembrane protein containing a serine/threonine kinase module and an endoribonuclease domain, executes site-specific cleavage of XBP1 mRNA to remove a 26-nucleotide intron during UPR. XBP1S is more potent as a transcriptional activator and more stable than XBP1U (unspliced). XBP1S activates the promoters of many genes, including those coding for enzymes necessary for the degradation of improperly folded ER proteins, and participates in cell proliferation and differentiation [53,54].

Firstly, our work also supports the concept that XBP1S is a key downstream molecule of BMP2 in chondrogenesis and endochondral bone growth based on the following evidence. (*i*) Both BMP2 and XBP1S are potent in inducing *in vitro* chondrogenesis and induction of chondrogenetic markers such as collagen II, collagen X and RUNX2 (Fig.3). The effect of XBP1S on chondrogenesis is similar to the effects of BMP2 and differs significantly from many factors that have opposite effects on collagen II and collagen X [40,47,48]; (*ii*) BMP2 induced XBP1S in chondrocytes, as shown in (Fig.5); (*iii*) Notably, knockdown of XBP1S strongly inhibited BMP2-mediated chondrogenesis, as assayed by collagen II, SOX9, collagen X and RUNX2 expression in the course of chondrocyte differentiation (Fig.5); (*iv*) Finally, BMP2 activates XBP1S specific reporter genes through Smad transcription factors (Fig.6).

Granulin-epithelin precursor, as a growth factor, has been linked to development, tissue regeneration, tumourigenesis and inflammation [26,31,55,32,56]. We previously reported that GEP accelerates chondrocyte hypertrophy, mineralization and endochondral bone growth through Erk1/2 signalling and its target gene, including JunB transcription factor [21]. Herein, we present evidence showing that XBP1S induces GEP expression in the course of chondrocyte development (Fig.7), and endogenous GEP is required for XBP1S-stimulated chondrocyte hypertrophy, mineralization and endochondral bone formation. Our previous report showing that (*i*) XBP1S cannot improve chondrocyte differentiation and endochondral bone formation in GEP^−/−^ BMSC cells, and (*ii*) XBP1S recovered the potent stimulating effect of chondrocyte differentiation, mineralization and endochondral bone growth in GEP null growth plates rescued by GEP (Fig.8). In addition, XBP1S increases GEP-mediated chondrocyte maturation, mineralization and endochondral bone growth. XBP1S enhances chondrocyte differentiation and endochondral bone formation through activating the chondrogenic activity of GEP (Fig.9).

Recently, we reported that ADAMTS-7 binds to and degrades COMP and that COMP interacts with GEP and potentiates GEP-stimulated chondrocyte functions, indicating that ADAMTS-7, GEP and COMP form an interaction and interplay network in regulating chondrocyte functions [57–59]. It remains to be determined how the interaction network among ADAMTS-7 metalloproteinase, GEP growth factor and COMP extracellular matrix molecule acts in concert in regulating chondrocyte differentiation and endochondral ossification. Our current study focuses on the relationship between XBP1S transcription factor, a UPR signal molecule, and GEP growth factor in chondrocyte development for the first time.

**Figure 10:**
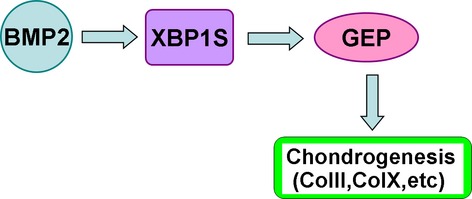
Proposed model for explaining the role and regulation of XBP1S in chondrocyte differentiation. XBP1S, induced by BMP2, stimulates chondrocyte differentiation by activating chondrogenic activity of granulin-epithelin precursor growth factor.

On the basis of the data in the literature [3,7,9,39], our earlier findings [21,22,46] and the results of this study, we propose a model for the role of XBP1S – specifically, its expression and function – in chondrocyte differentiation (Fig.10). This study provides novel insights into the role of XBP1S, a novel mediator in the BMP2 pathway, in regulating chondrocyte differentiation and endochondral bone formation and sheds light on the molecular mechanism by which XBP1S positively regulates chondrogenesis; *i.e*. XBP1S, a key downstream molecule of BMP2, increases chondrocyte differentiation and endochondral bone formation through activating GEP growth factor, and endogenous GEP is required for XBP1S-stimulated chondrocyte hypertrophy, mineralization and endochondral bone growth. Our work supports a hypothesis that XBP1S, a transcription factor induced by BMP2, regulates chondrogenesis and endochondral bone formation through GEP growth factor. The elucidation of XBP1S's role and molecular events involved in chondrocyte differentiation will better our understanding of normal cartilage development and the pathogenesis of cartilage disease.
